# The CHASIT study: sequential chemo-immunotherapy in patients with locally advanced urothelial cancer – a non-randomized phase II clinical trial

**DOI:** 10.1186/s12885-023-10963-7

**Published:** 2023-06-13

**Authors:** V. C. Rutten, Y. Salhi, G. J. Robbrecht, R. de Wit, G. J.L.H. van Leenders, T. C.M. Zuiverloon, J. L. Boormans

**Affiliations:** 1 Department of Urology, Erasmus MC Cancer Institute, Rotterdam, the Netherlands; 2 Department of Medical Oncology, Erasmus MC Cancer Institute, Rotterdam, the Netherlands; 3 Department of Pathology, Erasmus MC Cancer Institute, Rotterdam, the Netherlands

**Keywords:** Bladder cancer, Chemotherapy, Immunotherapy, Pathological response

## Abstract

**Background:**

Patients with locally advanced irresectable or clinically node positive urothelial cancer (UC) have a poor outcome. Currently, these patients can only be cured by receiving induction chemotherapy and, if an adequate radiological response is obtained, radical surgical resection. Long-term survival, however, strongly depends on the absence of residual tumor in the surgical resection specimen, i.e. a pathological complete response (pCR). The reported pCR rate following induction chemotherapy in locally advanced or clinically node-positive UC is 15%. The 5-year overall survival rate for patients achieving a pCR is 70–80% versus 20% for patients who have residual disease or nodal metastases. This clearly demonstrates the unmet need to improve clinical outcome of these patients. Recently, the JAVELIN Bladder 100 study demonstrated an overall survival benefit of sequential chemo-immunotherapy in patients with metastatic UC. The CHASIT study aims to translate these findings to the induction setting by assessing the efficacy and safety of sequential chemo-immunotherapy in patients with locally advanced or clinically node-positive UC. In addition, patient biomaterials are collected to investigate biological mechanisms of response and resistance to chemo-immunotherapy.

**Methods:**

This multicenter, prospective phase II clinical trial includes patients with stage cT4NxM0 or cTxN1-N3M0 UC of the bladder, upper urinary tract or urethra. Patients who do not experience disease progression after 3 or 4 cycles of platinum-based chemotherapy are eligible for inclusion. Included patients receive 3 cycles of anti-PD-1 immunotherapy with avelumab followed by radical surgery. Primary endpoint is the pCR rate. It is hypothesized that sequential chemo-immunotherapy results in a pCR rate of ≥ 30%. To obtain a power of 80%, 64 patients are screened and 58 patients are included in the efficacy analysis. Secondary endpoints are toxicity, postoperative surgical complications, progression-free, cancer-specific and overall survival at 24 months.

**Discussion:**

This is the first study to assess the potential benefit of sequential chemo-immunotherapy in patients with locally advanced or node positive UC. If the primary endpoint of the CHASIT study is met, i.e. a pCR rate of ≥ 30%, a randomized controlled trial is foreseen to compare this new treatment regimen to standard care.

**Trial registration:**

NCT05600127 at Clinicaltrials gov, registered on 31/10/2022.

**Supplementary Information:**

The online version contains supplementary material available at 10.1186/s12885-023-10963-7.

## Background

Urothelial cancer (UC) is the 10th most common cancer worldwide [1]. The current standard of care for patients with non-metastatic muscle invasive bladder cancer (MIBC) is cisplatin-based neoadjuvant chemotherapy followed by removal of the bladder (radical cystectomy, RC) and pelvic lymph node dissection (LND) [2]. Patients with muscle invasive, locally advanced irresectable or clinically node-positive disease (stage cT4NxM0 or cTxN1-N3M0) have a poor prognosis. Due to the extent of affected lymph nodes, patients are ineligible to undergo surgery. However, curative treatment by radical resection is still possible, given that patients respond adequately to treatment with induction chemotherapy. The absence of residual tumor in the surgical resection specimen is defined as a pathological complete response (pCR), which corresponds with a good prognosis. Patients who achieve a pCR have an excellent outcome with reported 5-yr overall survival rates of 70–80% [3]. Nevertheless, only 15% of locally advanced irresectable or clinically node-positive patients achieve a pCR [3]. In contrast, patients with residual muscle invasive disease (≥ ypT2) or nodal metastases (> ypN0) have 5-yr overall survival rates of 15–20% [4]. Therefore, there is a clear unmet need to increase the pCR rate and improve outcome of patients with locally advanced or clinically node-positive MIBC.

Pre-operative immunotherapy for MIBC is currently investigated in several clinical trials, either as monotherapy or concurrent with chemotherapy [5–9]. Neoadjuvant monotherapy with three cycles pembrolizumab in patients with stage cT2-3bN0M0 UC (n = 50) resulted in a pCR of 42%, whereas after neoadjuvant treatment with atezolizumab, 31% of cT2-4N0M0 patients (n = 95) achieved a pCR [5, 6]. Trials which administer both chemotherapy and immunotherapy usually do this concurrently instead of sequentially. In the AURA trial, patients were enrolled in distinct cohorts according to their cisplatin eligibility. The cisplatin eligible patients were treated with avelumab combined with cisplatin-gemcitabine or ddMVAC. Treatment with cisplatin-gemcitabine resulted in a pCR rate of 32% (n = 9) compared to 43% (n = 12) after ddMVAC [10]. In the cisplatin ineligible cohort, patients received either paclitaxel-gemcitabine and avelumab or avelumab alone. After treatment with paclitaxel-gemcitabine and avelumab, 7% of patients (n = 2) achieved pCR, in contrast to 25% (n = 7) after treatment with avelumab only [11]. However, these trials merely focus on the neoadjuvant setting and include patients with localized and lymph-node negative MIBC only.

In patients with locally advanced irresectable or node-positive UC, treatment with ipilimumab and nivolumab resulted in a pCR of 46% at interim analysis [12]. The Javelin Bladder 100 trial reported promising results with the sequential treatment of chemo- and immunotherapy. Patients with locally advanced or metastatic MIBC who had at least stable disease after platinum-based systemic chemotherapy, were treated with avelumab maintenance therapy [13]. This resulted in prolonged median overall survival and progression free survival compared to best supportive care after 2 years follow-up (23.8 vs. 15.0 months and 5.5 vs. 2.1 months, respectively) [14]. The present study aims to translate these beneficial outcomes of sequential chemo-immunotherapy to patients with locally advanced or node-positive UC. This study is the first to assess sequential chemo-immunotherapy in the induction and non-metastatic setting of UC.

The CHASIT study aims to improve the pCR rate and clinical outcome in patients with locally advanced irresectable or node-positive UC of the bladder, upper urinary tract or urethra. Patients with at least stable disease after treatment with platinum-based induction chemotherapy will receive 3 cycles of avelumab, an anti-PD-L1 antibody, followed by radical surgery. We hypothesize that sequential chemo-immunotherapy is more effective in obtaining pCR compared to induction chemotherapy alone, which results in improved clinical outcome.

## Methods

### Clinical trial design

**Fig. 1:**
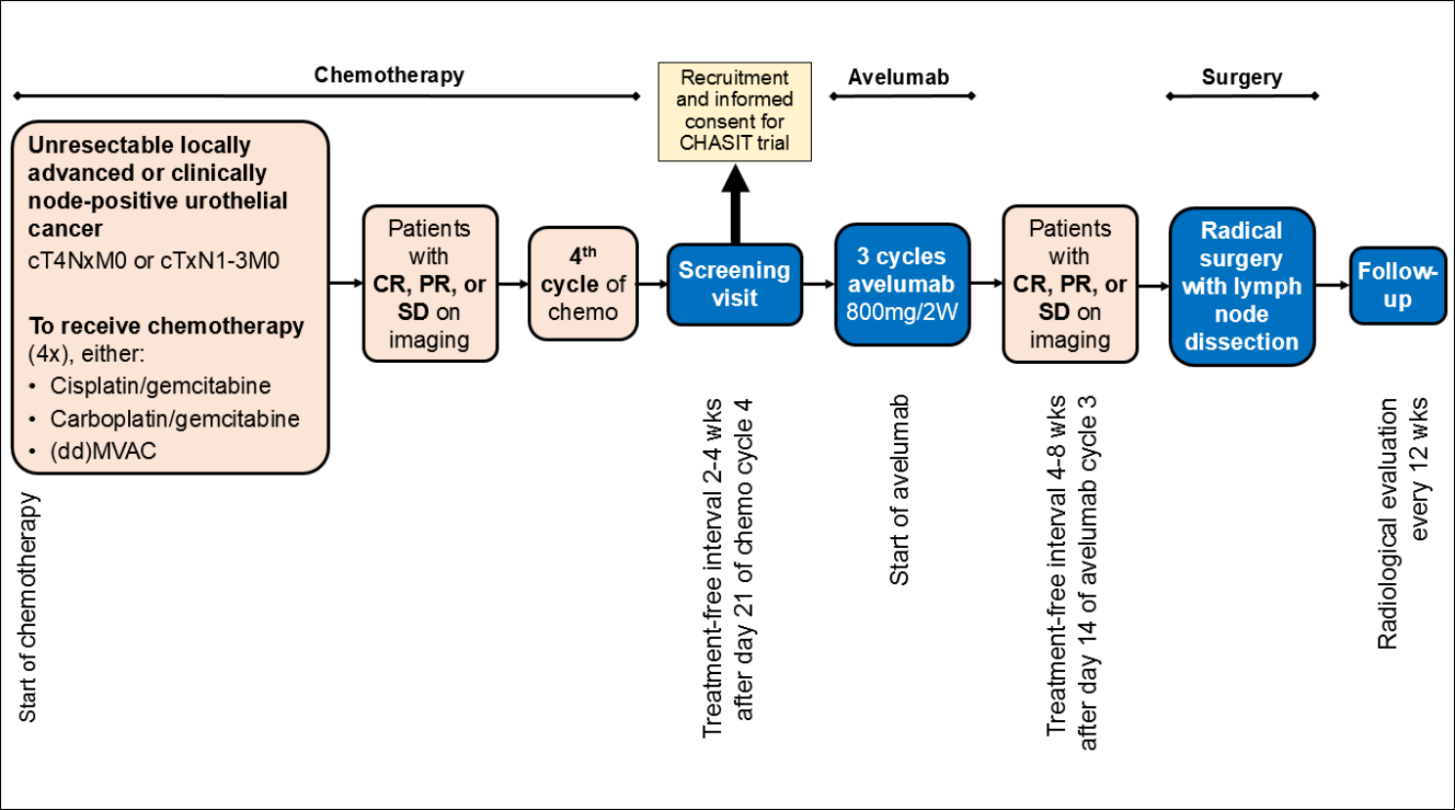
Flowchart of study design. Abbreviations: CR = complete response, PR = partial response and SD = stable disease

The CHASIT trial is a prospective, multicenter, non-randomized single arm phase II clinical trial. The following Dutch hospitals are participating: Erasmus Medical Center in Rotterdam, Amphia hospital in Breda, Radboud University Medical Center in Nijmegen and Jeroen Bosch hospital in Den Bosch. Patients with at least stable disease after treatment with platinum-based chemotherapy (gemcitabine + cisplatin, gemcitabine + carboplatin or (dd)MVAC) are eligible for inclusion and will be treated with avelumab. After avelumab treatment, response evaluation is performed with radiological imaging. In the absence of disease progression, patients are scheduled for surgical excision of the primary tumor and lymph nodes (Fig. 1). The study has been approved by the Medical Ethical Committee of the Erasmus University Medical Centre (MEC 2022 − 0257) on 10/10/2022, and has been registered at Clinicaltrials.gov (NCT05600127) on 31/10/2022.

### Objectives and endpoints

The main objective of this study is to improve the pathological response rate with sequential chemo-immunotherapy. The primary endpoint is the pCR rate, defined as the proportion of patients without residual disease in the surgical resection specimen (ypT0N0, *carcinoma in situ* is allowed) in the intention-to-treat analysis. Secondary end points are progression-free, cancer-specific and overall survival at 24 months, calculated from the time of first administration of avelumab, safety and tolerability of avelumab, surgical complications within 30 and 90 days from the date of surgery, the rate of pathological non-invasive residual UC in the resection specimen (< ypT2N0) and the proportion of patients in whom radical surgery is delayed > 8 weeks after the last administration of avelumab due to immune-related toxicity. All adverse events are graded according to the National Cancer Institute Common Terminology Criteria for Adverse Events (NCI-CTCAE version 5.0) [15]. Surgical complications are graded according to Clavien-Dindo classification system for surgical complications [16].

### Patient population

Eligible patients must have histologically confirmed UC of the bladder, upper urinary tract or urethra, clinical stage cT4NxM0 or cTxN1-N3 M0, and had at least stable disease after a minimum of 3 or a maximum of 4 cycles of platinum-based induction chemotherapy. A maximum of 50% divergent differentiation of histological subtypes is permitted. Clinical staging is based upon bimanual examination under anesthesia, computerized tomography (CT) with or without Positron Emission Tomography (PET) scan. Resection of the disease with curative intent should be possible and is assessed at a multidisciplinary board meeting. Patients must be fit and willing to undergo surgery. WHO performance status 0–2 and adequate bone marrow, renal and liver function are required. Further details on inclusion and exclusion criteria can be found in Appendix 1.

### Procedures

#### Screening and response evaluation before study entry

Induction chemotherapy should have been platinum-based (gemcitabine + cisplatin (gemcitabine 1000 mg/m^2^, day 1 and day 8, cisplatin 70 mg/m^2^, day 1, 3 week interval) or gemcitabine + carboplatin (gemcitabine 1000 mg/m^2^, day 1 and day 8, carboplatin AUC 4.5, day 1, 3 week interval) or (dd)MVAC (methotrexate 30 mg/m^2^, day 1, vinblastine 3 mg/m^2^, day 2, doxorubicin 30 mg/m^2^, day 2, and cisplatin 70 mg/m^2^, day 2, 2 week interval, with G-CSF), no other regimens are allowed. After 3 cycles of chemotherapy, treatment response is assessed radiologically by thoracoabdominal (PET-)CT-scan. Response evaluation is based on RECIST v1.1 criteria [17]. Patients are discussed at a multidisciplinary tumor board meeting. Patients who have at least stable disease and are eligible for study inclusion. Patients are screened for eligibility and undergo physical examination and laboratory tests at the outpatient department. They are asked to provide written informed consent for study participation. If eligible, subjects are scheduled for sequential treatment with avelumab and radical surgery thereafter.

#### Treatment with avelumab

Patients receive a maximum of 3 cycles avelumab every 2 weeks, provided that toxicity is acceptable. Avelumab is supplied by Merck (CrossRef Funder ID: 10.13039/100009945), as part of an alliance between Merck and Pfizer. The first administration should be within 2–4 weeks after day 21 of the last cycle of chemotherapy. Prior to each cycle, blood and urine is examined. Avelumab is given intravenously in 1 h at a dosage of 800 mg. Pre-medication is mandatory and is performed as per manufacturer’s recommendation to mitigate infusion-related reactions. Adverse events related to immunotherapy are to be managed according to local guidelines. Dose reduction for toxicity management is not permitted. In case of persisting toxicity, the next cycle of avelumab administration may be omitted until toxicity is resolved or decreased to at least Grade 1 in severity according to the NCI-CTCAE criteria [15]. A maximum dose interruption of 2 weeks is allowed. Toxicity management guidelines are provided and include actions such as decreasing the infusion rate, symptomatic treatment of symptoms or discontinuation of avelumab treatment, depending on the severity of toxicity.

#### Radical surgery

After the last cycle of avelumab, patients are re-staged by thoracoabdominal CT. If there are no signs of disease progression as per RECIST v1.1 criteria [17], patients are scheduled for radical surgery. Surgery must be performed within 4 to 8 weeks after day 14 of the last cycle of avelumab. Surgical procedures are dependent upon the location of the primary tumor: in case of UC of the bladder, radical surgery consists of an anterior exenteration (female) or radical cystoprostatectomy (male), in case of UC of the upper urinary tract a radical nephro-ureterectomy or a radical urethrectomy in case of UC of the urethra. All surgical procedures are combined with an extended LND: pelvic LND in case of UC of the bladder, retroperitoneal LND in case of UC of the upper urinary tract and inguinal LND for UC of the urethra. Surgery is performed by a urologist and the surgical technique is at the discretion of the treating physician and may include open, laparoscopic or robot-assisted surgery.

#### Pathological review

All formalin-fixed, paraffin-embedded (FFPE) tissue specimens are reviewed centrally by an experienced uropathologist (GvL). The presence of residual cancer cells is assessed, as well as tumor grade (WHO 1973 and 2004/2016), the presence and proportion of divergent differentiation and urothelial subtypes, lymphovascular invasion, concomitant CIS, surgical margins status, the number of resected lymph nodes and lymph node metastasis, as well as the presence of extranodal extension.

#### Follow-up

Patients who continued to surgery after completion of ≥ 1 cycle of avelumab will have regular follow-up visits every 3 months until 2 years after study inclusion, including blood withdrawal and radiological examination. In case of a recurrence or disease progression, a diagnostic confirmatory tissue biopsy is performed. In case of disease progression, all subsequent treatment modalities applied will be recorded until 2 years after surgery. In addition, included patients who did not receive avelumab will be followed as well as part of the intention-to-treat analysis.

### Biomarker analysis

Exploratory studies are planned to investigate biological mechanisms of response and resistance to chemo-immunotherapy. Findings will be correlated to clinical outcome in order to identify predictive biomarkers that allow the selection of patients who benefit most likely from immunotherapy. Biopsy and surgical tumor specimens pre- and post-immunotherapy are collected, as well as in case of disease progression during follow-up. In addition, blood and urine samples are collected at baseline, during avelumab treatment, prior to surgery and during follow-up.

### Statistical analysis

Historically, the pCR rate following induction chemotherapy in patients with cT4NxM0 or cTxN1-N3M0 UC is 15% [3]. We hypothesize that the pCR in the study is equal to or higher than 30%. A one sample proportion test is used to compare the historical rate to the study arm. Assuming an alpha of 2.5%, 53 patients are required to obtain a power of 80%. Assuming a dropout rate of 10% and a screening failure rate of 10%, 64 patients need to be screened.

An interim analysis to assess efficacy and safety is planned after inclusion of the first 40 patients (75% of the total accrual). If the pCR in the first 40 subjects is less than 15%, the study will terminate prematurely. A data safety monitoring committee (DSMB) was installed to ensure the safety of the participants and the validity and integrity of safety data generated from the study. The study is open for inclusion since December 2022 and the estimated final inclusion date is December 2024. All data will be prospectively collected.

## Discussion

Maintenance therapy with avelumab has shown to be well-tolerated and beneficial for survival in metastatic MIBC patients who had at least stable disease after systemic chemotherapy [13, 14]. We aim to translate these findings to patients with locally advanced or clinically node-positive disease. This is the first trial to assess the efficacy of sequential chemo-immunotherapy in this group of patients. We aim to test the hypothesis that sequential treatment with chemo-immunotherapy is effective, safe and does not cause a delay in surgical treatment. In addition, collected biomaterials will provide a basis for future exploratory studies on possible biomarkers of response and resistance to chemo-immunotherapy. The findings of this trial will contribute to understanding, improving and predicting clinical outcome for patients with locally advanced or node-positive UC.

Chemotherapy in the neoadjuvant setting is effective to obtain a pathological complete response. Neoadjuvant treatment of cT2-T4aN0M0 patients with MVAC increased the pCR rate from 15% after cystectomy alone to 38% [18]. Neo-adjuvant treatment with ddMVAC resulted in higher pCR rates and longer 3-year progression-free survival compared to neoadjuvant cisplatin and gemcitabine treatment in cT2-T4a patients (42% vs. 36% and 66% vs. 56%, respectively) [19]. In cT3-T4a patients, 37% of patients (n = 1013) achieved complete pathological downstaging to non-muscle invasive disease (≤(y)pT1N0) after neoadjuvant chemotherapy (20). The CHASIT study, however, includes patients with more advanced disease, representing a different patient population than the neoadjuvant setting. Therefore we expect that pCR rates will be lower compared to the neoadjuvant setting. Literature on this specific and relatively rare population is limited. In a cohort of 52 patients with histologically proven cN1-3 disease, 29% of patients achieved pCR after treatment with MVAC (21). This high pCR rate in lymph node-positive patients can be explained due to the inclusion of 11 patients with cN1 disease: these patients formally belong to the neoadjuvant setting (instead of induction setting). They are expected to respond better to NAC compared to cN2-3 patients, which causes an elevated pCR rate. There was no pCR rate reported for the cN2-3 subgroup (21). A larger cohort of patients with cT1N1-3M0 or cT2-4aN0-3M0 disease (n = 304) was treated with various regimens of induction chemotherapy: MVAC, gemcitabine + cisplatin or other regimens (gemcitabine + carboplatin, other carboplatin based regimens and taxanes). After a median of 4 cycles administered, the reported pCR rate was 15% [3]. Due to the limited number of other studies available, this study was selected as a historical cohort. The CHASIT study aims to translate the findings of the Javelin Bladder 100 trial and therefore only patients with response or stable disease after treatment with platinum-based chemotherapy are eligible for inclusion. In the Javelin Bladder 100 trial, patients received 4 to 6 cycles of chemotherapy and switched thereafter to avelumab maintenance therapy. In the CHASIT study, the last 2 cycles of chemotherapy are replaced by 3 cycles of immunotherapy.

Although ideally a randomized controlled trial (RCT) is the preferred study design, an RCT would necessitate a large number of subjects to be included, making it logistically and financially challenging to conduct this trial. This does not seem feasible on a national level. Therefore, we decided to conduct a single arm phase II study. If the CHASIT study shows that sequential chemo-immunotherapy leads to a pCR rate of ≥ 30%, an international randomized controlled trial is foreseen to compare this new treatment regimen to standard care.

## Electronic supplementary material

Below is the link to the electronic supplementary material.

Supplementary Material 1

## Data Availability

The datasets generated and/or analyzed during the current study are not publicly available due to the fact that data sill have to be generated and the current manuscript contains the study protocol only. Once data collection has finished, data will be available from the corresponding author on reasonable request.

## Abbreviations

CT: Computerized tomography
DSMB: Data safety monitoring board
FFPE: Formalin-fixed, paraffin-embedded
MIBC: Muscle-invasive bladder cancer
pCR: Pathological complete response
LND: Lymph node dissection
PET: Positron emission tomography
RC: Radical cystectomy
RCT: Randomized controlled trial
UC: Urothelial carcinoma
