# Supplementary material for: The CHASIT study: sequential chemo-immunotherapy in patients with locally advanced urothelial cancer – a non-randomized phase II clinical trial
Source: BMC Cancer. 2023 Jun 13;23:539. doi: 10.1186/s12885-023-10963-7 (PMC10262374; doi:10.1186/s12885-023-10963-7)
Supplement: Supplementary file 1 — Supplementary Material 1 [file 12885_2023_10963_MOESM1_ESM.docx]

**Appendix 1. List of inclusion and exclusion criteria**

# Inclusion criteria The following subjects are eligible for the present study:

1. Age ≥ 18 years.
2. Have histologically confirmed urothelial carcinoma of the bladder, upper urinary tract or urethra; a maximum of 50% divergent differentiation or histological subtypes is permitted.
3. Have clinical stage cT4NxM0 or cTxN1-N3M0 as assessed by bimanual examination under anaesthesia, CT scan, MRI scan or PET-CT scan.
4. Have at least stable disease after a minimum of 3 or a maximum of 4 cycles of induction chemotherapy with Cisplatin / Carboplatin + Gemcitabine according to RECIST v1.1.
5. Are fit and willing to undergo radical surgery with removal of lymph node template including all affected lymph nodes and the primary tumor.
6. World Health Organisation performance status of 0-2.
7. Provide written informed consent.
8. Negative pregnancy test in women with childbearing potential.
9. Adequate bone marrow function, including:
10. Absolute neutrophil count (ANC) ≥1,500/mm^3^ or 1.5 x 10^9^/L;
11. Platelets ≥100 x 10^9^/L;
12. Hemoglobin ≥ 5.6 mmol/L (may have been transfused).
13. Adequate renal function, defined as estimated creatinine clearance ≥30 mL/min as calculated by the CKD-EPI eGFR.
14. Adequate liver function, including:
15. Total serum bilirubin ≤ 1.5 x upper limit of normal (ULN);
16. Aspartate aminotransferase (AST) and alanine aminotransferase (ALT) ≤ 2.5 x ULN.

# Exclusion criteria A potential subject who meets any of the following criteria will be excluded from participation in this study:

1. Predominant (>50%) non-urothelial carcinoma histology in the diagnostic endoresection specimen of the bladder, urethra or upper urinary tract.
2. Any test for hepatitis B virus (HBV) or hepatitis C virus (HCV) indicating acute or chronic infection.
3. Have an estimated creatinine clearance as assessed by the CKD-EPI eGFR of <30 ml/min.
4. Prior exposure to immune-mediated therapy with exclusion of *Bacillus-Calmette Guérin* intravesical instillations, including but not limited to other anti-CTLA-4, anti PD-1, anti PD-L1, or anti-PD-L2 antibodies.
5. Persisting toxicity related to prior chemotherapy (Grade >2 NCI CTCAE v5.0).
6. A diagnosis of any other malignancy within 2 years prior to inclusion, except for adequately treated basal cell or squamous cell skin cancer or *carcinoma in situ* of the breast or of the cervix, low grade prostate cancer on surveillance without any plans for treatment intervention, or prostate cancer that has been adequately treated with prostatectomy or radiotherapy and currently with no evidence of disease.
7. ≤2 cycles of induction platinum-based chemotherapy received.
8. Progression of disease during or following induction platinum-based chemotherapy, as assessed by RECIST v1.1.
9. Distant metastatic disease.
10. Previous pelvic radiation therapy.
11. Breastfeeding women.
12. Bilateral upper urinary tract urothelial carcinoma.
13. Active autoimmune disease that might deteriorate when receiving an immuno-stimulatory agent. Patients with diabetes type I, vitiligo, psoriasis, or hypo- or hyperthyroid disease not requiring immunosuppressive treatment are eligible.
14. Any of the following in the previous 6 months: myocardial infarction, severe/unstable angina, coronary/peripheral artery bypass graft, symptomatic congestive heart failure, cerebrovascular accident, transient ischemic attack or symptomatic pulmonary embolism.
15. Active infection requiring systemic therapy.
16. Known severe hypersensitivity reactions to monoclonal antibodies (Grade 3), any history of anaphylaxis, or uncontrolled asthma (i.e. 3 or more features of asthma symptom control per the Global Initiative for Asthma 2015).
17. Known prior or suspected hypersensitivity to avelumab.
18. Current use of immunosuppressive medication, EXCEPT the following:
19. Intranasal, inhaled, topical steroids, or local steroid injections (e.g. intra-articular injection);
20. Systemic corticosteroids at (equivalent) doses of maximum 10 mg prednisone;
21. Steroids as premedication for hypersensitivity reactions (e.g. CT scan premedication).
22. Diagnosis of prior immunodeficiency or organ transplant requiring immunosuppressive therapy, or known human immunodeficiency virus (HIV) or acquired immunodeficiency syndrome (AIDS)-related illness.
23. Vaccination within 4 weeks of the first dose of study treatment and while on trial is prohibited except for administration of inactivate vaccines (e.g. inactivated influenza vaccines) or mRNA vaccines (e.g. COVID-19 vaccines).
24. Other severe acute or chronic medical conditions including colitis, inflammatory bowel disease, and pneumonitis; psychiatric condition including recent (within the past year) or active suicidal ideation or behaviour; or laboratory abnormality that may increase the risk associated with study participation or study treatment administration or may interfere with the interpretation of study results and, in the judgment of the investigator, would make the patient inappropriate for entry into this study.
